# Eosinophil granulocytes in chronic inflammatory respiratory diseases and CRSwNP: Function, immunological basis, and clinical significance 

**DOI:** 10.5414/ALX02469E

**Published:** 2024-03-21

**Authors:** Felix Klimek, Christoph Bergmann, Jan Hagemann, Mandy Cuevas, Sven Becker, Oliver Pfaar, Ingrid Casper, Ludger Klimek

**Affiliations:** 1 Center for Rhinology and Allergology, Wiesbaden,; 2 Practice for Ear, Nose and Throat disease, Clinic RKM 740, Düsseldorf,; 3 Department for Otolaryngology, University Medical Center Mainz, Mainz,; 4 Department for Otolaryngology, University Hospital Carl Gustav Carus, TU Dresden, Dresden,; 5 Department for Otolaryngology, University Hospital Tübingen, Tübingen, and; 6 Section of Rhinology and Allergy, Department of Otorhinolaryngology, Head and Neck Surgery, University Hospital Marburg, Philipps-Universität Marburg, Marburg, Germany

**Keywords:** eosinophils, mucositis, CRSwNP, asthma, allergic rhinitis, biologicals, mepolizumab, benralizumab, reslizumab

## Abstract

Introduction: Eosinophils play an important regulatory and immunomodulatory role in airway mucosa and have antiparasitic and antiviral properties as well as pro-inflammatory effects that may also cause persistence of inflammation with tissue remodeling. The number of eosinophils and the detection of specific mediators in biological samples from, e.g., blood, nasal secretions, and bronchial fluid can serve as biomarkers that reflect the underlying pathophysiology of certain diseases, predict treatment success, and detect therapy effects. Materials and methods: A literature search was conducted to determine the immunologic basis, mode of action, clinical significance, and available evidence for therapeutic approaches using eosinophil-targeted monoclonal antibodies by searching Medline, Pubmed, and the national and international trial database (ClinicalTrials.gov) and guideline registries as well as the Cochrane Library. Human studies published on the topic in the period up to and including 10/2023 were considered. Results: Based on the international literature and previous experience, the results are summarized, and recommendations are given. Conclusion: The important role of eosinophils in immunological processes in the airway mucosa is comprehensively analyzed and can serve as a basis for current and future treatment approaches.

## Introduction 

Eosinophils develop both beneficial and harmful activities as part of immune responses, they are involved in the maintenance of health and homeostasis, but also in the development of diseases. Their role in the pathophysiology of various allergic and non-allergic diseases has been known for decades [1, 2]. This has led to the development of a wide range of therapies focusing on eosinophils, either acting non-specifically by inhibiting various upstream or downstream immune pathways or specifically by eosinophil-targeted biologics [3, 4]. 

Recently, several previously unknown physiological functions of eosinophils have been identified, consisting of important regulatory and immunomodulatory, anti-inflammatory, antiparasitic, and antiviral properties [5, 6]. On the other hand, the involvement of pro-inflammatory eosinophils in the initiation, progression, and persistence of inflammation with tissue remodeling is well known and has been documented for many decades – especially in the upper and lower respiratory tract. Therefore, the eosinophil count in biological samples from different body compartments such as blood, nasal secretions, or bronchial fluid can serve as a biomarker that reflects the underlying pathophysiology of certain diseases, can roughly predict treatment success, and detect therapy effects [3, 4, 7]. The precise definition of eosinophilia and the distinction between an actual pathological condition and hypereosinophilia as an epiphenomenon are crucial for the correct interpretation and use of eosinophils as biomarkers in clinical practice. 

## Origin and life cycle 

Eosinophils are part of the innate immune system and belong to the white blood cell family [8]. These cells were first described by Paul Ehrlich in the 19^th^ century [9], who gave his name to the German regulatory authority in vaccines, the Paul Ehrlich Institut in Langen. Eosinophils have a characteristic bilobed nucleus and large granules that can be intensely stained with the dye eosin, which gave the cells their name. The granules contain several enzymes and cationic proteins, including peroxidases, lysosomal enzymes, and the major basic protein (MBP). Eosinophils originate from the bone marrow, where they are formed from a myeloid precursor that they share with basophils [10]. At the myelocyte stage, the precursor cells stop dividing and enter a maturation phase of ~ 4 days, during which the cells mature into functional granulocytes [11]. 

This process is regulated by cytokine receptors (e.g., CD131-containing receptors: CD116/CD131, CD123/CD131, and CD125/CD131, which bind to GM-CSF, IL-3, and IL-5, respectively [12]), alarmin receptors (e.g., ST2, which binds to IL-33) [13], and specific transcription factors (e.g., GATA1/2 and C/EBPs) [14]. Subsequently, mature eosinophils are released from the bone marrow and can be detected in small numbers in the peripheral blood (~ 50 – 150 cells/mL blood; 1 – 3% of total leukocytes) in homeostasis [15]. The possibility of in situ eosinophilopoiesis has also been described [16]. In homeostasis, the half-life of eosinophils in peripheral blood is unknown but is estimated to be 11 – 63 hours [17, 18, 19]. 

In a healthy state, eosinophils can be detected in various tissues such as the intestine and fatty tissue, where they fulfill various homeostatic functions. An increased number of pre-activated eosinophils can be found in the peripheral blood and in inflamed target tissues in various diseases, especially but by no means only in allergic diseases [20]. In addition to the classic allergic respiratory diseases asthma and rhinitis, which are associated with eosinophil infiltration of the target organs, there is a broad spectrum of non-allergic diseases (e.g., non-allergic eosinophilic asthma and eosinophilic bronchitis, eosinophilic granulomatosis with polyangiitis, chronic rhinosinusitis with nasal polyps (CRSwNP), non-allergic eosinophilic rhinitis, eosinophilic esophagitis (EoE), anaphylaxis, etc.) [21, 22]. etc.) [21, 22, 23, 24, 25, 26, 27, 28, 29, 30, 31, 32, 33]. They can be accompanied by high eosinophil counts, which can be detectable both in the blood and in the tissue. 

IL-5 is a central cytokine for the life cycle of eosinophils as it is firstly a growth factor for eosinophil progenitor cells, secondly is involved in the mobilization of eosinophils from the bone marrow, and thirdly plays an important role in their activation and their colonization in target tissues [34]. However, the presence of IL-5 is not solely crucial for the development of eosinophils, as IL-5 knockout mice still exhibit activated eosinophils [35]. 

A study with a monoclonal antibody against IL-5 (mepolizumab) showed a significant decrease in eosinophils in the peripheral blood and inflamed tissue in patients with EoE but had no effect on the eosinophil count in the duodenum [36]. Similarly, treatment with mepolizumab significantly reduced the number of eosinophils in peripheral blood and sputum [37], but did not lead to a significant reduction in eosinophils in airway tissue and eosinophil activation markers such as MBP [38]. This may be due to the fact that a certain subset of airway resident eosinophils do not respond to IL-5 [39]. There is a growing consensus that IL-5 plays an important role in reactive eosinophilia, whereas it appears to be less important for homeostatic eosinophils in tissues. A detailed and comprehensive overview of all migration and activation factors of eosinophils as well as their mediators and receptors can be found in a recent review [40]. 

## Functions 

Traditionally, eosinophils have been described as important cells of the innate immune defense against multicellular parasites, especially helminths. This is largely based on observations of eosinophilia in the context of parasitic diseases and the killing of parasites by eosinophils and their toxic granules in vitro [41]. 

However, profound functional mechanisms in which eosinophils are involved are only partially understood yet and may also differ from species to species, as eosinophils only make a variable contribution to parasite killing in mouse models, for example [42]. One of the most important tasks of eosinophils is to maintain tissue homeostasis in various organs such as the lungs, nose and paranasal sinuses, gastrointestinal tract, thymus, and adipose tissue. They support the normal function of the immune system (immune tolerance), promote fertility and prevent obesity and bronchial hyperreactivity [43, 44, 45]. This is mainly due to the fact that these eosinophils are found in numerous healthy human tissues (see overview) [6] and in mouse models of metabolism [46], endometriosis [47], and other tissue functions [48]. The mechanisms underlying these functions are discussed in more detail below. 

## Mechanisms of eosinophil activation and function 

Eosinophils express a variety of receptors on their surface, which have been described in detail in a recent review [8]. Several receptors are important for therapeutic targeting of specific eosinophil-related diseases. Eosinophils express three receptors with a common β-chain (GM-CSF, IL-3 and IL-5 receptor), all of which are involved in the control of the eosinophil life cycle, including survival. In addition, the eosinophil-specific Siglec-8 is also involved in their survival. Several eosinophil receptors are involved in adhesion to the endothelium (for example, L-selectin, Mac-1/CD11b/CD18, VLA-4/CD49d) and chemotaxis (for example, CCR3/CD193, C5aR/CD88, platelet activation receptor) [49]. In addition, several surface receptors are associated with eosinophil activation (for example, Fcγ RII/CD32A, Fcα R/CD89, CR3 - CD11b, glucan receptors) [40, 50, 51, 52, 53]. 

Eosinophils are highly cytotoxic due to their intracellular mediators, which can become a potential threat to the host tissue if they are not adequately controlled. Pre-activation or priming is a crucial mechanism in this process. Under homeostatic conditions, eosinophils tend to be refractory cells that can only respond to high concentrations of activators, such as opsonized particles. However, when eosinophils are exposed to specific cytokines or other immune mediators in vitro, even at low concentrations, they can rapidly (within 1 – 15 minutes, depending on the priming agent) transition to a pre-activated phenotype that makes them highly susceptible to these targets [20, 54]. Priming can also occur in patients with eosinophil-mediated diseases [55]. 

Eosinophils have a whole arsenal of cytotoxic effector mechanisms that are mainly exerted extracellularly, i.e., within the synapse between the cell and its major (i.e., parasitic) targets. These functions include the abundant production of reactive oxygen species by a membrane-bound NADPH oxidase (NOX-2) [56], the degranulation of highly cytotoxic granular proteins (for example, MBP, eosinophil peroxidase, eosinophil cationic protein (ECP), or eosinophil-derived neurotoxin (EDN)) [57] or peptides (e.g., polycationic peptides) into the synapse and the killing of extracellular targets by the formation of eosinophilic extracellular traps (EET) [58]. In addition, eosinophils produce a variety of cytokines (e.g. IL-4, IL-5, and GM-CSF) and bioactive lipid mediators (e.g., leukotriene C4 and platelet activating factor), which are released upon activation [59, 60, 61]. 

Eosinophils are relatively inert or refractory in the bloodstream and in tissues. When activated by receptors, they release their cytotoxic components and cause tissue damage. Eosinophils harbor a unique secretory organelle, the so-called crystalloid granule, which contains MBP in high concentrations and leads to the formation of a crystalline core. The content of the crystalloid granules can only be released by eosinophils through degranulation. There are several types of degranulation in eosinophils, most of which fall into the category of classical exocytosis with SNARE-mediated membrane fusion (including compound exocytosis and piecemeal degranulation, the latter occurring mainly in allergic inflammation) [60, 62, 63, 64, 65, 66]. In addition, free eosinophil granules can be released as intact, membrane-bound organelles by a form of necrotic release also known as cytolysis [67]. Eosinophils also release DNA into the extracellular space during EET formation. The molecular mechanism of this process is not yet fully understood [58, 68]. EET formation occurs independently of degranulation, although granule proteins have been detected on DNA strands [69]. The association of granule products with DNA has been suggested to occur both before [58, 70] and after their release [71]. EETs have been shown to increase the viscosity of nasal secretions in patients with chronic rhinosinusitis [72]. In addition, EETs have also been associated with Charcot-Leyden crystals, which in turn have been associated with eosinophilia in the past [73]. Furthermore, eosinophil-derived Charcot-Leyden crystals in the mucus of asthma patients play a role in allergic inflammation, goblet cell metaplasia, IgE synthesis, and bronchial hyperreactivity [74]. 

In addition to their involvement in the defense against parasites, eosinophils are also involved in other aspects of immunity as outlined below. 

### Antiviral functions 

Eosinophils can inactivate viruses. Granular proteins of eosinophils such as ECP and the neurotoxin EDN have RNAse activity that can inhibit viral replication in situ. This antiviral effect may be lost in patients with allergic asthma [64] and this may explain why viral infections often precede exacerbations of allergic asthma. Eosinophils possess several pathogen-related receptors that can recognize viral antigens (for example, toll-like receptors TLR-3, -7, -9 and RIG-I receptor), produce several cytokines with antiviral effects (for example, IL-2, IL-12, and IFN-γ), express costimulatory molecules (for example, CD80, CD86, CD28, CD40) and are actively involved in the presentation of viral antigens to CD8 T lymphocytes [75]. Possible defense mechanisms of eosinophils against SARS-CoV-2 in COVID-19 have also been described [76]. While eosinopenia has been identified as a prospective screening, diagnostic, and prognostic tool, the actual role of eosinophils in lung pathology in this infection is unclear [77]. Eosinopenia has been shown to be an early, appropriate diagnostic and screening tool for COVID-19 infection [78, 79] and a prognostic marker for disease severity and unfavorable outcome in patients with COVID-19 pneumonia [80, 81, 82, 83]. Interestingly, eosinophilia (especially in asthma patients treated with inhaled corticosteroids) has been associated with a better outcome of COVID-19 disease [84]. On the other hand, studies analyzing the outcome of COVID-19 in patients with severe asthma under biologic therapy showed inconsistent results – also regarding SARS-CoV-2 vaccinations [85, 86, 87, 88, 89, 107]. 

### Other anti-infective effects of eosinophils 

The prominent role of eosinophils in parasitic infections is well known. These effects include antigen presentation and modulation of the T-cell reaction. They modulate the production of IgE and the production of mucus by goblet cells. In addition, their granular proteins are directly involved in the killing and neutralization of parasites [42, 90]. On the other hand, eosinophils can also have a deleterious effect in certain parasitic infections, which can contribute to tissue damage [91]. Eosinophils also play a role in the complex defense against selected bacteria. Although their phagocytic activity and bacterial killing is lower than that of neutrophils, they contribute to the elimination of selected bacteria, while their granular proteins and enzymes help to neutralize bacterial proteins [92, 93]. The formation of extracellular traps by eosinophils (stimulated by various mediators, for example, thymic stromal lymphopoietin) is an important phenomenon in bacterial killing [94]. Finally, eosinophils also exert antifungal activities. They use their CD11b surface receptor to recognize β-glucan – a major component of the cell wall of fungi [53]. Proteases released by fungi activate protease-activated receptors in eosinophils, resulting in the release of various cytokines. In addition, eosinophils can probably inactivate fungal spores [95]. 

### Modulation of inflammation and fibrosis 

It is often not known that eosinophils also have regulatory or even anti-inflammatory properties. In mast cell-induced inflammation, for example, they can modulate the harmful effects of mast cell activation by oxidative deamination of histamine and enzymatic inactivation of other mast cell inflammatory mediators [96]. Eosinophils can also suppress T cells (“regulatory eosinophils”) [97]. In fibrogenesis, eosinophils play a pathophysiological role by releasing TGF-β and thus stimulate collagen production by parenchymal cells [98]. 

## Hypereosinophilic syndrome (according to Schwaab et al. [99]) 

Blood eosinophils are measured by taking a differential blood count. For better comparability, it should be carried out under standardized conditions and without recent physical stress. A distinction is made between relative and absolute hypereosinophilia: the limit values of 0.5 G/lL or 6% in the differential blood count from whole blood apply here. Blood hypereosinophilia can also be graded from mild to moderate to severe and in terms of its duration – transient, episodic, persistent. In order to make a diagnosis of hypereosinophilia in the blood, two abnormal samples must be taken more than 2 weeks apart. 

Hypereosinophilia can also be detected in organs other than the blood. Tissue hypereosinophilia is diagnosed by clinical-pathological assessment of tissue samples if eosinophils or their markers are found extensively in the tissue (bone marrow: > 20% of nucleated cells). Tissue damage is not necessarily present in this case; however, if this is the case, a diagnosis of (tissue-specific) hypereosinophilic syndrome (Schwaab et al. [99]) should be made, depending on whether or not blood hypereosinophilia is also present at the same time. 

Eosinophilia is associated with a variety of diseases that have different underlying causes and can affect different organs [99]. The diagnostic approach to hypereosinophilia is facilitated by the established subdivision into primary and secondary (= reactive) hypereosinophilia [100], which have been further refined according to updated classifications [101, 102]. Recently, new, refined diagnostic criteria and a classification of eosinophilic diseases have been proposed [103]:

- Familial (hereditary) hypereosinophilia – is often diagnosed in childhood and is sometimes associated with immunodeficiencies;
- Hypereosinophilia of unknown significance – without familial clustering, underlying pathology, associated molecular (genetic) abnormalities or organ damage caused by hypereosinophilia;
- Secondary (reactive) hypereosinophilia – non-clonal eosinophilia due to various immunoregulatory mechanisms;
- Primary (clonal, neoplastic) hypereosinophilia – caused by malignant degenerated eosinophils.

## Conclusion 

In addition to the well-characterized pro-inflammatory and disease-promoting effects of eosinophils in the context of chronic inflammatory diseases – including in the upper and lower respiratory tract – these cells also have homeostatic, anti-inflammatory, and anti-infectious activities. 

These have often not been adequately considered to date, but these properties must be taken into account when selecting therapies that affect eosinophils in different ways. Therapeutic effects can range from a reduction in the number of cells to the complete depletion of eosinophils. 

It is important to note that there has been considerable progress in therapies targeting eosinophils and that these therapies have become much more targeted and precise due to the availability of eosinophil (IL-5)-targeting monoclonal antibodies (mAbs, biologics) [104, 105, 106]. 

All biologics approved to date have shown a positive treatment effect and improved disease burden in patients with eosinophil-related diseases. However, studies on predictors of a good clinical response to these biologics are still insufficient and, particularly in patients with severe eosinophilic disease, there appears to be involvement and simultaneous activation of multiple signaling pathways. Phenotyping patients based on eosinophil granulocytes in the blood may therefore not be accurate enough for targeted endotyping. In CRSwNP and also in asthma, for example, the use of blood eosinophils as the only biomarker often proved to be insufficient for selecting the right drug for the right patient or for efficient monitoring of therapeutic response. 

Therefore, it is important to understand the underlying immunology of CRSwNP and possibly establish immunoendotyping of patients to select the most appropriate treatment. 

## Funding 

Funding for this article lay on unrestricted educational grant from German Allergy Society AeDA. 

## Conflict of interest 

J. Hagemann received grants from Novartis Pharma GmbH, GlaxoSmithKline Deutschland GmbH, Sanofi, HAL Allergy for advisory boards and lectures outside the submitted work. 

M. Cuevas has received honoraria from ALK-Abelló, Allergopharma, AstraZeneca, Bencard Allergie/ Allergy Therapeutics, GalaxoSmithKline, HAL Allergy, Leti Pharma, Novartis, Roxall, Sanofi-Aventis, Stallergenes outside the submitted work and memberships in the following organizations: AeDA, DGHNO. 

L. Klimek has received research grants and/or lecture honoraria from Allergy Therapeutics/Bencard, UK/Germany; ALK-Abelló, Denmark; Allergopharma, Germany; ASIT Biotech, Belgium; AstraZeneca, Sweden, Bionorica, Germany; Biomay, Austria, Boehringer Ingelheim, Germany, Circassia, USA; Stallergene, France; Cytos, Switzerland; Curalogic, Denmark; HAL, Netherlands; Hartington, Spain; Lofarma, Italy; MEDA/Mylan, Sweden/USA; Novartis, Switzerland, Leti, Spain; ROXALL, Germany; GlaxoSmithKline (GSK), UK; Sanofi, France and/or acted as a consultant for the above-mentioned pharmaceutical companies. Dr. Klimek is editor of Allergo Journal and Allergo Journal International and is the current President of AeDA (Medical Association of German Allergists) and Vice President of EAACI. 

O. Pfaar reports grants and/or personal fees from ALK-Abelló, Allergopharma, Stallergenes Greer, HAL Allergy Holding B.V./HAL Allergie GmbH, Bencard Allergie GmbH/Allergy Therapeutics, Lofarma, ASIT Biotech Tools S.A., Laboratorios LETI/LETI Pharma, GlaxoSmithKline, ROXALL Medizin, Novartis, Sanofi-Aventis and Sanofi-Genzyme, Med Update Europe GmbH, streamedup! GmbH, Pohl-Boskamp, Inmunotek S.L., John Wiley and Sons, AS, Paul-Martini-Stiftung (PMS), Regeneron Pharmaceuticals Inc., RG Aerztefortbildung, Institut für Disease Management, Springer GmbH, AstraZeneca, IQVIA Commercial, Ingress Health, Wort&Bild Verlag, Verlag ME, Procter&Gamble, ALTAMIRA, Meinhardt Congress GmbH, Deutsche Forschungsgemeinschaft, Thieme, Deutsche AllergieLiga e.V., AeDA, Alfried-Krupp Krankenhaus, Red Maple Trials Inc., Königlich Dänisches Generalkonsulat, Medizinische Hochschule Hannover, ECM Expro&Conference Management, Technische Universität Dresden, Lilly, Paul Ehrlich Institut (PEI), Japanese Society of Allergy, Forum für Medizinische Fortbildung, Dustri Verlag, all outside the submitted work and within the last 36 months; and he is member of EAACI Excom, member of ext. board of directors DGAKI; coordinator, main- or co-author of different position papers and guidelines in rhinology, allergology, and allergen-immunotherapy and Associate Editor of the journal(s) Allergy and Clinical Translational Allergy (CTA). 

S. Becker has received honoraria for lectures, consulting and research from ALK Abéllo, Allergopharme, HAL Allergie, Bencard Allergie, Allergy Therapeutics, Therma Fisher Scientific, Novartis, AstraZeneca, Sanofi Genzyme, GSK, MSD, Mylan, Viatris, Karl Storz GmbH, Altamira, Auris Medical, Smart Reporting, Stryker, Helix Biopharm, BMBF. 

C. Bergmann, I. Casper, and F. Klimek have no conflict of interest in connection with this work. 
